# Improved time-varying reproduction numbers using the generation interval for COVID-19

**DOI:** 10.3389/fpubh.2023.1185854

**Published:** 2023-06-30

**Authors:** Tobhin Kim, Hyojung Lee, Sungchan Kim, Changhoon Kim, Hyunjin Son, Sunmi Lee

**Affiliations:** ^1^Department of Applied Mathematics, Kyung Hee University, Yongin, Republic of Korea; ^2^Department of Statistics, Kyungpook National University, Daegu, Republic of Korea; ^3^Department of Preventive Medicine, College of Medicine, Pusan National University, Busan, Republic of Korea; ^4^Busan Center for Infectious Disease Control and Prevention, Pusan National University Hospital, Busan, Republic of Korea; ^5^Department of Preventive Medicine, College of Medicine, Dong-A University, Busan, Republic of Korea

**Keywords:** COVID-19, latent period, generation interval, incubation period, serial interval, presymptomatic transmission

## Abstract

Estimating key epidemiological parameters, such as incubation period, serial interval (SI), generation interval (GI) and latent period, is essential to quantify the transmissibility and effects of various interventions of COVID-19. These key parameters play a critical role in quantifying the basic reproduction number. With the hard work of epidemiological investigators in South Korea, estimating these key parameters has become possible based on infector-infectee surveillance data of COVID-19 between February 2020 and April 2021. Herein, the mean incubation period was estimated to be 4.9 days (95% CI: 4.2, 5.7) and the mean generation interval was estimated to be 4.3 days (95% CI: 4.2, 4.4). The mean serial interval was estimated to be 4.3, with a standard deviation of 4.2. It is also revealed that the proportion of presymptomatic transmission was ~57%, which indicates the potential risk of transmission before the disease onset. We compared the time-varying reproduction number based on GI and SI and found that the time-varying reproduction number based on GI may result in a larger estimation of $R_{t}$, which refers to the COVID-19 transmission potential around the rapid increase of cases. This highlights the importance of considering presymptomatic transmission and generation intervals when estimating the time-varying reproduction number.

## 1. Introduction

Owing to the spread of coronavirus disease 2019 (COVID-19), the World Health Organization declared COVID-19 a pandemic on March 11, 2020. As a result of the widespread transmission of the virus, governments and public health organizations around the world have implemented various interventions to slow the spread of the virus and mitigate its impact on public health. These interventions include measures such as social distancing, mask-wearing and lockdowns, as well as efforts to increase testing and contact tracing. The effectiveness of these interventions is often evaluated by tracking the basic reproduction number ($R_{0}$) of the virus, which represents the average number of secondary infections caused by a single infected individual. Accurate estimates of $R_{0}$ are essential for predicting the course of the pandemic and informing public health decision-making. However, estimating $R_{0}$ is challenging, particularly in the early stages of an outbreak, when data is limited and key epidemiological parameters are uncertain.

The timing of transmission plays a crucial role in the spread and control of an epidemic, as well as its relationship to the basic reproduction number $R_{0}$ and the growth rate of the outbreak. The serial interval (SI) is the time between the onset of symptoms in a primary case (i.e., the infector) and a secondary case (i.e., the infectee) that is observable (1, 2). The generation interval (GI) is the time between the infection in an infector and the infection in an infectee (3, 4). These parameters such as latent period and GI are important but often unknown. Understanding the time interval between infection in an infector and an infectee is crucial. Additionally, the incubation period, which is the time between infection and the onset of symptoms, is important for individual treatment, while the latent period is important for disease transmission. When infectiousness starts after symptom onset, the serial interval is used to approximate the generation time (5, 6). Presymptomatic transmission can occur before symptoms appear if the latent period is shorter than the incubation period or if the infection occurs before symptoms appear (3, 7).

Previous studies showed that the distribution of generation times and the incubation period is estimated simultaneously (1, 3, 8). The mean latent period was estimated to be 5.5 days, which is shorter than the mean incubation period (6.9 days) (9). Lau et al. (1) provided a statistical method to jointly estimate the generation time and incubation period from 80 human-to-human transmission pairs in China. The generation time and incubation period were estimated to be 5.7 and 4.8 days, respectively. Wu et al. (6) explored the impact of variants on the incubation periods. In a total of 142 studies with 8,112 patients, the mean incubation period of COVID-19 was 5.00 days for cases caused by the Alpha variant, 4.50 days for those caused by the Beta variant, 4.41 days for those caused by the Delta variant and 3.42 days for those caused by the Omicron variant. Mcaloon et al. (10) estimates the serial interval and proportion of presymptomatic transmission as 4.0 days and 0.67, respectively, based on contact tracing data in Ireland.

Furthermore, the basic reproduction number $R_{0}$ is investigated in Wang et al. (11), Zhang et al. (12), and Leveau et al. (13). Various approaches using compartmental models, statistical models and Bayesian analysis were employed to investigate COVID-19 transmission dynamics (14–16). The basic reproduction number $R_{0}$ is a summary measure of the transmission potential of an infectious disease during an early epidemic (5, 17). The time-dependent reproduction number $R_{t}$ represents the instantaneous reproduction number, which refers to the expected number of secondary infections caused by an infector at a specific point in time. An $R_{t}$ value greater than 1 suggests that the disease is spreading, while an $R_{t}$ value <1 indicates that the disease is being controlled through interventions (17, 18).

The aim of our study was to simultaneously determine the probability distribution of crucial epidemiological parameters by utilizing data from 72 infector-infectee pairs during the outbreak of COVID-19 in Busan, South Korea. Additionally, we used the maximum likelihood method to estimate important epidemiological parameters related to transmission dynamics, such as the latent period, serial interval, incubation period and generation interval. The proportion of presymptomatic transmission was also estimated to indicate the potential risk before the onset of symptoms. We also compared the time-dependent reproduction numbers based on SI and GI using a statistical model and evaluated the potential risk of using GI distribution instead of SI distribution in regards to presymptomatic transmission. These estimated parameters were used to improve the understanding of the transmissibility of COVID-19 and the effectiveness of interventions during the various stages of the outbreak.

## 2. Materials and methods

### 2.1. Data

**Figure 1:**
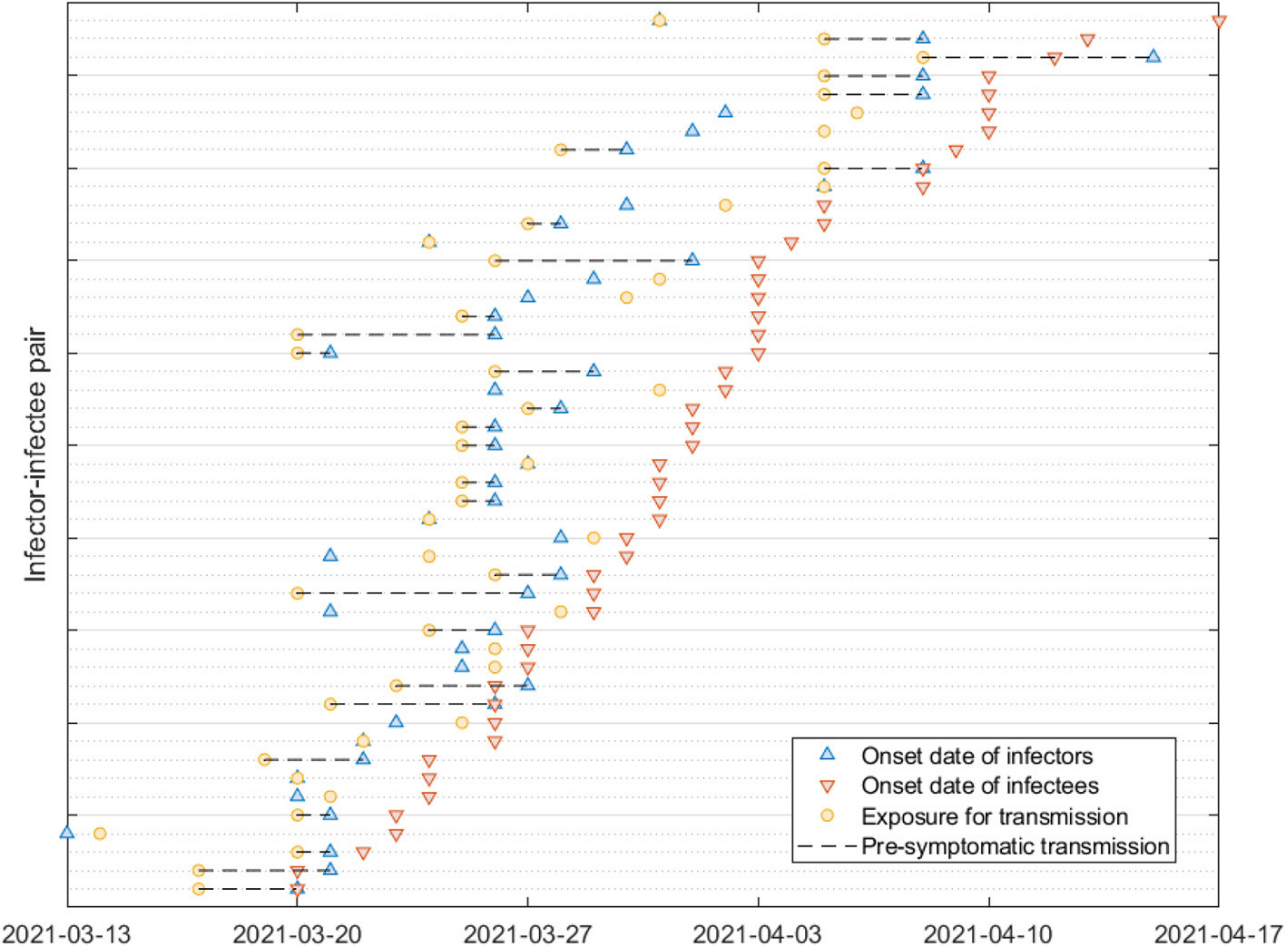
Some selected infector-infectee pairs are displayed. Triangles indicate the date of onset of symptoms in infectors (blue) and infectees (red). The circles indicate the exposure of the infectee to the infector (yellow). Black dashed lines indicate transmission of presymptomatic cases.

We employ a total of 144 cases of 72 infector-infectee pairs collected by epidemiological investigators in Busan, South Korea, between February 2020 and April 2021. The data includes the time of infection in infectees and the onset of symptoms in both infectors and infectees and was used to study key epidemiological parameters related to single exposure events. Some selected infector-infectee pairs of the last month are shown in Figure 1, which illustrates a subset of the pairs, with blue triangles indicating when the infector showed symptoms, red inverted triangles indicating when the infectee showed symptoms, yellow circles indicating when the infectee was infected and dotted lines indicating cases of infection before symptoms appeared (which refers to presymptomatic transmission). Note that in April 2021, the Delta variant accounted for four cases within the metropolitan area and 42 cases through external transmission, making up 1.6% of the total cases under national variant surveillance. It wasn't until May that Busan and Gyeongsangnam-do began detecting the variant at a level of around 0.2%, and by July, it had reached 38.5% (19). Due to the absence of perfect surveillance for variant viruses among all COVID-19 cases in Korea, it is challenging to determine the exact number of individual cases caused by each variant. Since our data collection in Busan only extended until April 2021, we can infer that the number of cases attributable to the Delta variant is negligible.

**Figure 2:**
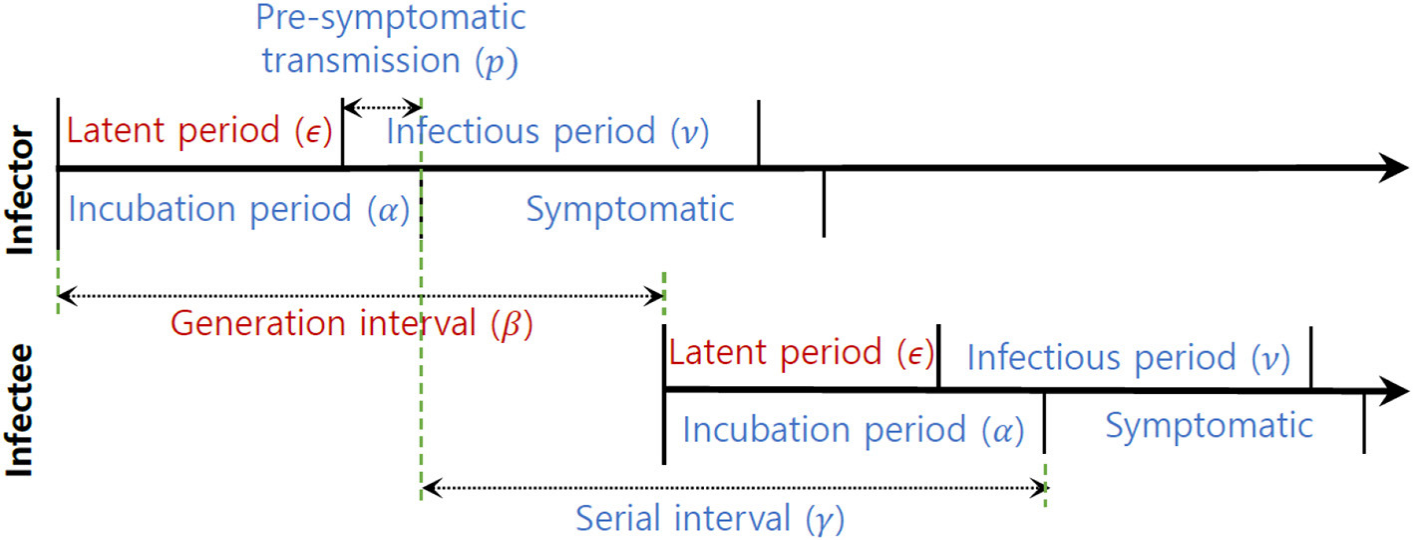
The upper arrow denotes an infector and the lower arrow denotes an infectee. Notation for some key parameters are shown: *α* (incubation period), *β* (generation interval), *γ* (serial interval), *p* (presymptomatic transmission), ν (infectious period), and *ϵ* (latent period).

Figure 2 provides a visual representation of the terms related to the transmission process between an infector (denoted by *o*) and infectee (denoted by *e*), including the incubation period (*α*), generation interval (*β*), serial interval (*γ*), latent period (*ϵ*) and the proportion of presymptomatic transmission (*p*). Moreover, Table 1 presents the mean and standard deviation of these parameters, calculated from the 72 infector-infectee pairs in the surveillance data. Specifically, the mean incubation period was found to be 5.2 days (with a standard deviation of 3.4 days). The 1st and 3rd quartiles were 3,7 and the 95th percentile was 11.9. The serial interval had a mean of 4.6 days and a standard deviation of 3.7 days. The 1st and 3rd quartiles were 2,7 and the 95th percentile was 10.9. The proportion of presymptomatic transmission was 54.2% of all cases.

**Table 1:** Epidemiological data of the 72 infector-infectee pairs.

**Empirical data**	**Mean**	**Median**	**SD**	***Q*_1_**	***Q*_3_**	**95th percentile**	**Unit**
Incubation period	5.2	4	3.4	3	7	11.9	Day
Serial interval	4.6	4	3.7	2	7	10.9	
Proportion of presymptomatic transmission	54.2						%

### 2.2. Estimation of serial and generation intervals

In this section, we estimated the incubation period, generation interval (GI) and serial interval (SI). We assumed that the incubation period and generation interval followed a gamma distribution. A gamma distribution is commonly assumed for numerous positive and continuous random variables (time-related distributions) as reported in many studies (3, 20, 21). More details of the methods given below are found in Zhao et al. (3). First, the incubation period is represented by *α*. Here, *α*_*o*_ and *α*_*e*_ represent the incubation period of the infector and infectee, respectively, in a transmission relationship. We assume that the distribution of the incubation period for infectors and infectees is identical and that *α*_*o*_ and *α*_*e*_ are independent and have the same probability density function *f*_*α*_ as a gamma distribution. For parametric distributions with a mean *μ*_*α*_ and a standard deviation of *σ*_*α*_, the log-likelihood function to be maximized is denoted by *l*_*α*_:


(1) $$\begin{array}{l} l_{\alpha }\left(\mu_{\alpha },\sigma_{\alpha }|{\text{Data}}^{ip}\right)=\overset{N_{ip}}{\underset{i=1}{\sum }}\log f_{\alpha }\left({\text{Data}}_{i}^{ip}|\mu_{\alpha },\sigma_{\alpha }\right) \end{array}$$

where ${\text{Data}}_{i}^{ip}$ is the observed incubation period of the *i*th infectee and *N*_*ip*_ is the number of observations of the incubation period. Next, we estimated the GI *β*. We assume follows a certain parametric distribution with the probability density *f*_*β*_, mean *μ*_*β*_ and SD *σ*_*β*_. The following equation was obtained:


(2) $$\begin{array}{l} \gamma =\beta +\alpha_{e}-\alpha_{o}, \end{array}$$

where *γ* is SI. According to the Equation (2), a probability density function of SI (*γ*), *f*_*γ*_, can be derived as follows:


$$\begin{array}{l} f_{\gamma }\left(\gamma \right)=\underset{\mathbb{R}}{\int }f_{\beta }\left(\gamma -y\right)\left[\underset{\mathbb{R}}{\int }f_{\alpha }\left(z\right)f_{\alpha }\left(y+z\right)\text{d}z\right]\text{d}y, \end{array}$$

with the help of the independence of α_*e*_ and α_*o*_. The log-likelihood to estimate the associated parameters of *f*_*γ*_, *l*_*γ*_, is


(3) $$\begin{array}{l} l_{\gamma }\left(\mu_{\alpha },\sigma_{\alpha },\mu_{\beta },\sigma_{\beta }|{\text{Data}}^{si}\right)\\ \;=\overset{N_{si}}{\underset{j=1}{\sum }}\log f_{\gamma }\left({\text{Data}}_{j}^{si}|\mu_{\alpha },\sigma_{\alpha },\mu_{\beta },\sigma_{\beta }\right). \end{array}$$

where ${\text{Data}}_{j}^{si}$ is the observed SI between the *j*th infectee and *j*th infector and *N*_*si*_ is the number of observations of SI.

Finally, we solve *f*_*α*_ and *f*_*γ*_ jointly: the likelihood is


(4) $$\begin{array}{l} l\left(\mu_{\alpha },\sigma_{\alpha },\mu_{\beta },\sigma_{\beta }|{\text{Data}}^{ip}\cup {\text{Data}}^{si}\right)=l_{\alpha }+l_{\gamma }. \end{array}$$

### 2.3. Estimation of presymptomatic transmission and latent period

In this section, we estimated the proportion of secondary infections caused by presymptomatic transmission, represented by *p*. It is worth noting that this proportion is equivalent to ℙ(*γ* < *α*). To account for the relationship between the incubation period and serial interval, we used a bivariate distribution, which was calculated using the following covariance matrix


$$\begin{array}{l} \left[\begin{array}{cc} \sigma_{\alpha }^{2} & \text{cov}\left(\alpha ,\gamma \right)\\ \text{cov}\left(\alpha ,\gamma \right) & \sigma_{\gamma }^{2}. \end{array}\right]. \end{array}$$

We estimate *p* using Monte Carlo simulation. From the definition of presymptomatic transmission, we directly got the relationship


(5) $$\begin{array}{l} \mathbb{P}\left(\nu \leq \text{𝔼}\left[\alpha \right]-\text{𝔼}\left[\epsilon \right]\right)=p. \end{array}$$

Next, if we assume that the random variables latent period *ϵ*, infectious period ν, and infectivity are independent, we get the relation from Svensson (22):


(6) $$\begin{array}{l} \text{𝔼}\left[\beta \right]=\text{𝔼}\left[\epsilon \right]+\frac{\text{𝔼}\left[\nu^{2}\right]}{2\text{𝔼}\left[\nu \right]}. \end{array}$$

For example, if ν ~ Γ(*k*, θ), where *k* is a shape parameter and θ is a scale parameter, *𝔼*[*β*] = *𝔼*[*ϵ*] + (1 + *k*)θ/2. These relationships satisfy 0 < *ϵ* < min{*α*, *β*}. We infer the latent period with Equations (5, 6) by substituting the estimated mean of incubation period, GI and presymptomatic transmission proportion. Recall that incubation, infectious periods and generation intervals are assumed to be a gamma distribution. The gamma distribution is a two-parameter family of continuous random variables (κ and θ), where the relation between the two parameters determines the mean and variance. We have used Matlab for all simulations. First, the standard deviation of the incubation period was computed using a built-in fmincon. Secondly, a Hessian matrix is obtained, then maximum likelihood estimation (MLE) is used to compute the confidence interval of the incubation period. Similarly, the standard deviation of the generation interval was computed using a built-in fmincon. Secondly, a Hessian matrix is obtained, then MCMC is used to compute the confidence interval of the generation interval.

### 2.4. Time-dependent reproduction number

The instantaneous reproduction number, $R_{t}$, is calculated using the *EpiEstim* package on the R software, described in Thompson et al. (17), Koyama et al. (23), and Alvarez et al. (24). This package is the standard method to compute $R_{t}$ from $I_{t}=\overset{t}{\underset{\tau =1}{\sum }}I_{t-\tau }g_{\tau }$, where *I*_*t*_ indicates the number of COVID-19 cases in time *t* and *g*_τ_ is the distribution of SI or GI. In the present study, we estimated $R_{t}$ for COVID-19 in Busan, Korea and determined the proportion of presymptomatic transmission. The proposed method improve the basic reproduction number, inferring the effect of control intervention without assuming exponential growth of cases. Moreover, the time-varing reproduction numbers are compared according to distribution of the generation interval or serial interval for *g*_τ_, which are estimated from the pairs data of infected individuals and their infectors.

## 3. Results

**Figure 3:**
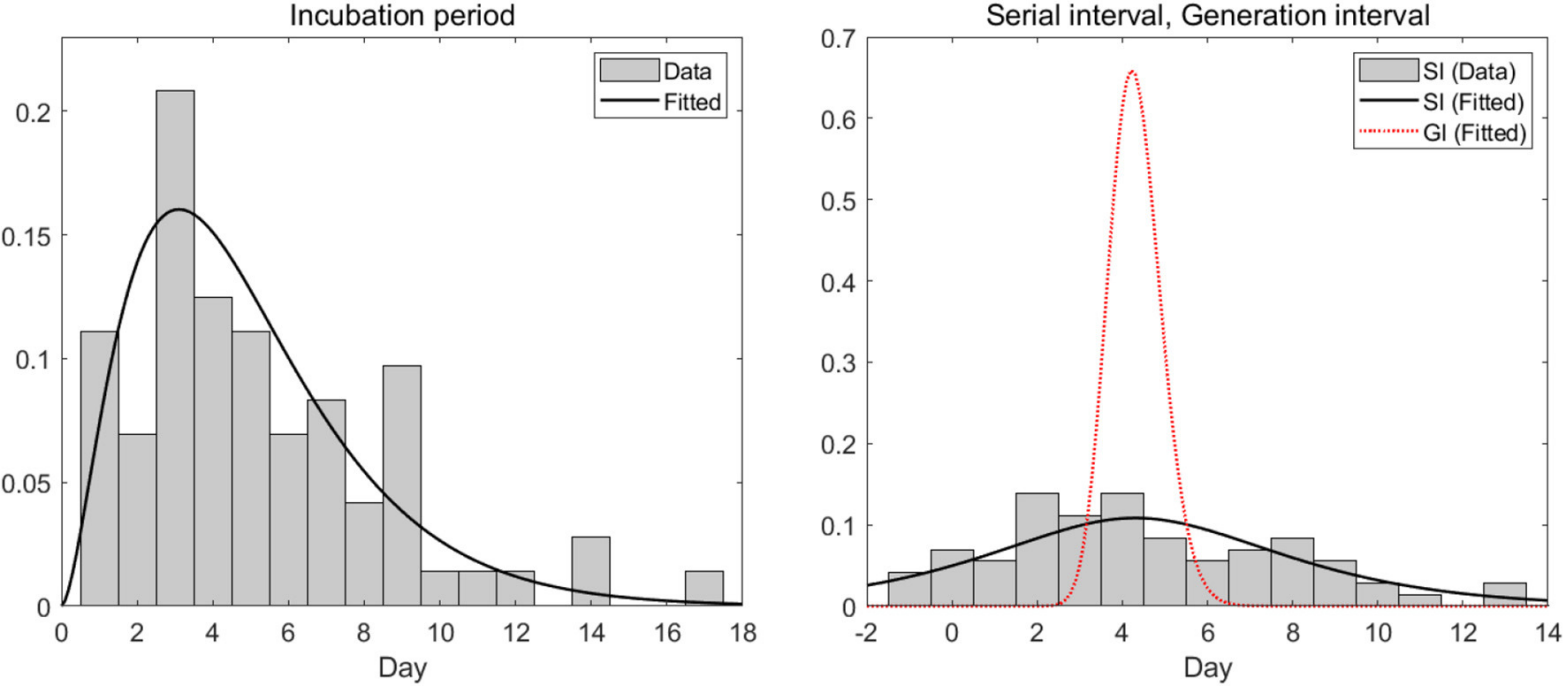
Empirical (observed) and estimated probability density distributions of the **(left)** incubation period and **(right)** serial interval (SI) and generation interval (GI).

In this section, we analyzed empirical data and estimated key parameters, as shown in Figure 3. Table 2 summarizes the mean and standard deviation (SD) of the estimated key parameters. The left side of Figure 3 illustrates the distribution of incubation periods, both from the observed data (bar) and the fitted distribution (solid curve). The mean and standard deviation of the observed incubation period were 5.2 days and 3.4 days, respectively, while the mean and standard deviation of the fitted incubation period were estimated to be 4.6 days (with a 95% confidence interval of 4.2–5.7) and 3.0 days (with a 95% confidence interval of 2.4–3.6), respectively. On the right side of Figure 3, we show the distributions of the serial interval and generation interval. We assumed that the GI follows a gamma distribution and used maximum likelihood estimation to obtain the fitted distribution. The mean GI was estimated to be 4.3 days (with a 95% CI of 4.2–4.4), with a standard deviation of 0.6 (with a 95% CI of 0.5–0.7), while the mean SI was estimated to be 4.3 days, with a standard deviation of 4.2. It is mathematically and epidemiologically reasonable that the standard deviation of SI is greater than that of GI as reported in Champredon et al. (25).

**Table 2:** Epidemiological parameters are estimated from the 72 selected pairs given in Table 1.

**Parameter**	**Notation**	**Mean**	**95% CI (Mean)**	**SD**	**95% CI (SD)**	**Unit**
Incubation period	*α*	4.9	4.2, 5.7	3.0	2.4, 3.6	Day
Generation interval	*β*	4.3	4.2, 4.4	0.6	0.5, 0.7	
Serial interval	*γ*	4.3		4.2		
Presymptomatic transmission	*p*	56.5				Percent (%)

**Figure 4:**
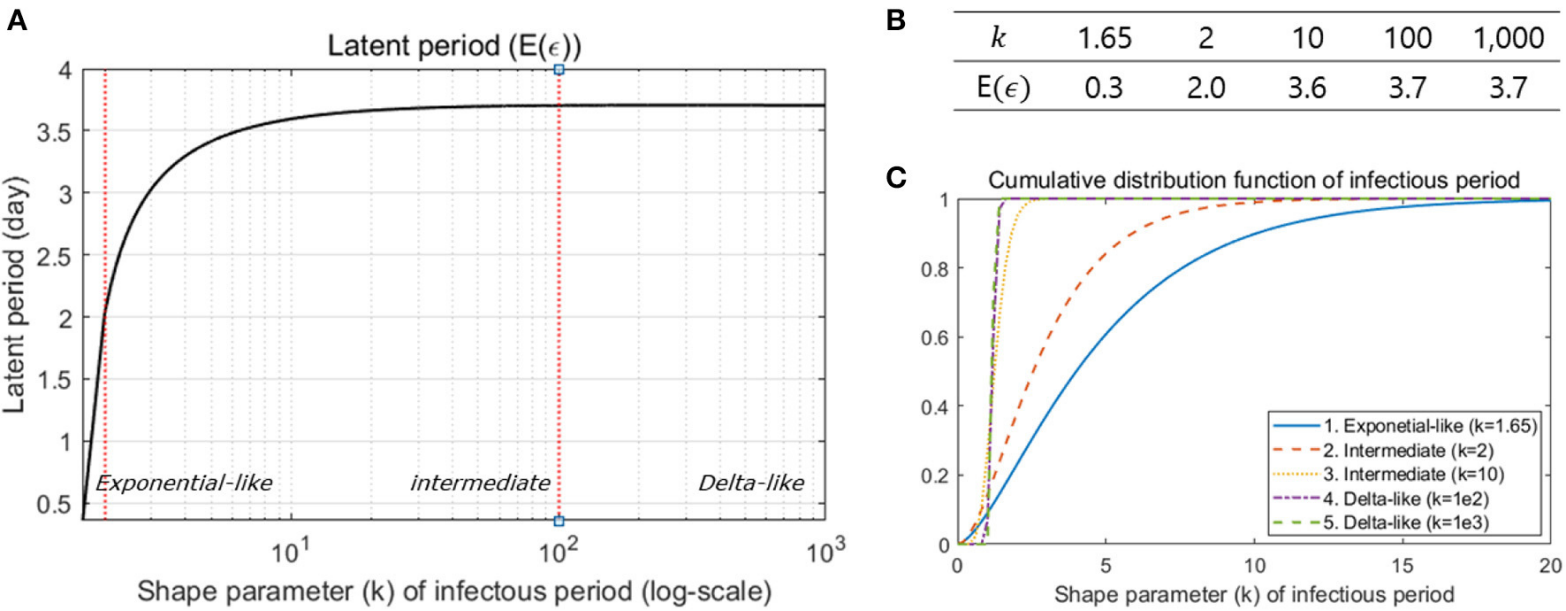
**(A)** The mean latent period is illustrated under different shape parameter values of the infectious period. **(B)** Some values are specified in the table. **(C)** Sensitivity analysis was performed under three different ranges of the shape parameter: exponential-like (*k* < 2), intermediate gamma (2 ≤ *k* < 100), and delta-like (*k*≥100).

Next, we analyzed 72 pairs of infected individuals and their infectors to determine the proportion of presymptomatic transmission. It was found that an empirical proportion 54.2% of transmission occurred before symptoms appeared and later this proportion was estimated to be 56.5%. We also examined the distribution of the latent period, using the estimated distribution of the incubation period, the generation interval and the proportion of presymptomatic transmission. The results indicated that the mean of the latent period became longer as the shape parameter of the infectious period increased as shown in Figure 4A, with an expectation of 0.3 days for exponential-like distribution, 2.0–3.7 days for intermediate gamma distribution and 3.7 days for delta-like distribution. Figure 4B shows the mean of the latent periods when *k* is 1.65, 2, 10, 100, and 1,000. Figure 4C is a plot of the cumulative distribution function of the infectious period according to the value of the shape parameter (*k*) of the infectious period. Because of the relation between the infectious period and latent period, the result occurs when *k*≥1.645. Particularly, *k* represents the shape parameter for the infectious period with a large value since we want to make sure that the mean of the latent period converges to a finite value (3.8 days) for any range of *k* as shown in Figures 4A, B.

**Figure 5:**
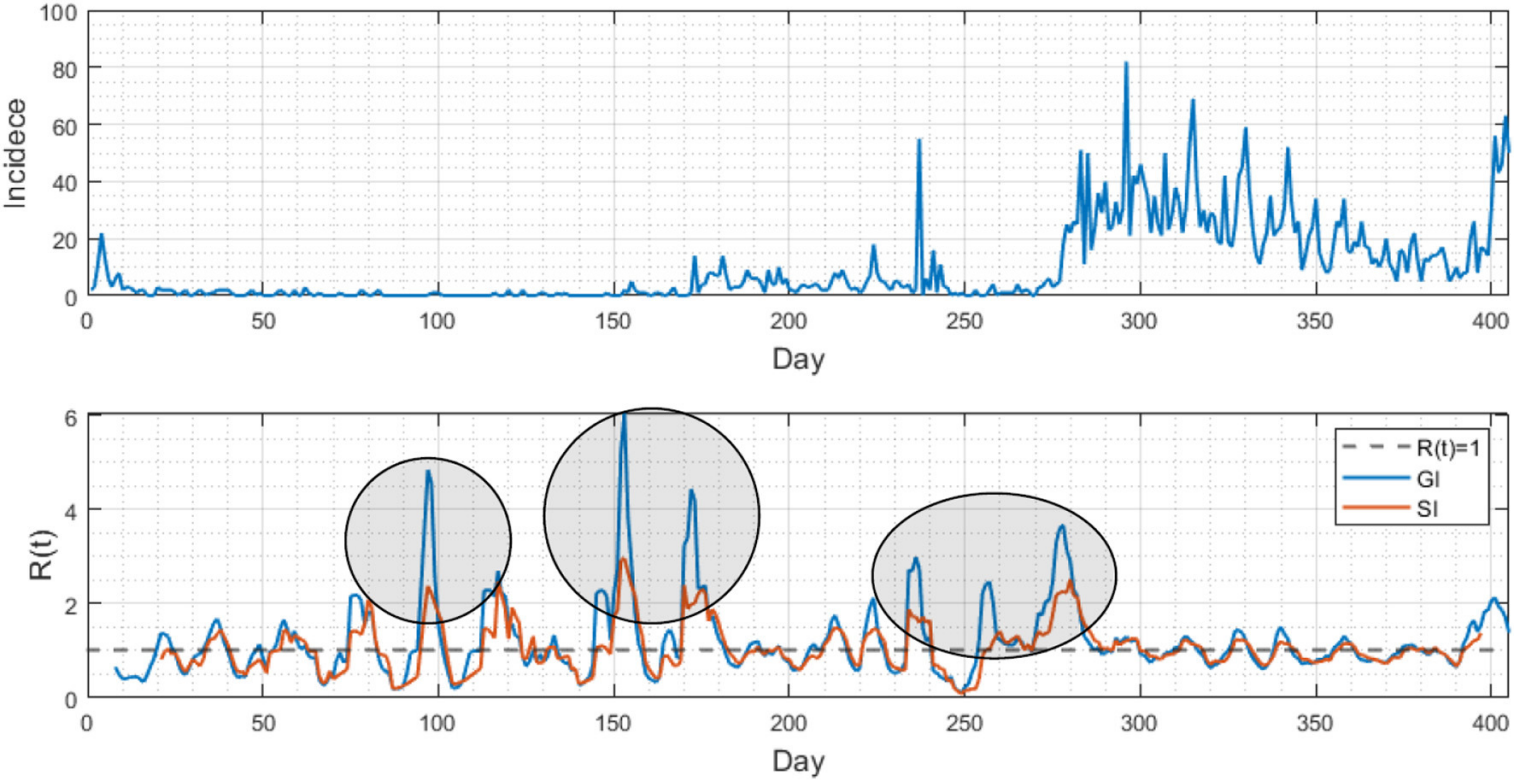
**(Upper)** Daily confirmed cases are displayed from February 2020 to March 2021 in Busan. **(Lower)** Two time-dependent reproduction numbers $R_{t}$ are compared using the generation interval (blue curve) and serial interval (red curve). Note that the $R_{t}$ results are distinct at some periods (see three circled periods).

Lastly, Figure 5 compared the differences in analyzing epidemic patterns using the generation interval and serial interval by displaying daily confirmed cases and the time-dependent reproduction number ($R_{t}$). The upper portion of the figure displays the daily confirmed cases in Busan, while the lower portion shows the estimated $R_{t}$ values for both GI (blue) and SI (red), calculated using a renewal model. The initial growth rate of the infection was determined to be 0.59. The results indicate distinct variations when comparing the values obtained from the GI and SI methods, with $R_{0}$ values of 3.7 and 11.9, respectively.

## 4. Discussion

We aimed to estimate key epidemiological parameters such as the incubation period, serial interval, generation interval and latent period for COVID-19 in South Korea, using infector-infectee surveillance data. We were able to implement the 3T (testing, tracing, and treating) strategy effectively, allowing us to collect data through contact tracing. Particularly, in the Busan region of Korea, we were able to obtain information on the initial and final exposures for most of confirmed cases. One of the key strengths of this data is the precise determination of the contact time. As a result, we were able to gather data on 72 pairs for our analysis. Previous research has also been conducted on data collection and analysis for the Busan region during the early stages of the COVID-19 outbreak in Son et al. (26).

Our results showed that the mean incubation period was estimated to be 4.9 days (95% CI: 4.2, 5.7), the mean generation interval was 4.3 days (95% CI: 4.2, 4.4) and the mean serial interval was 4.3 days with a standard deviation of 4.2. We also found that presymptomatic transmission accounted for a significant proportion of overall transmission, at around 50%. These estimates provide valuable information for understanding the transmission dynamics of COVID-19 and can aid in the development of more effective interventions and control measures.

To the best of our knowledge, this study is the first to estimate the latent period, generation interval, serial interval and proportion of presymptomatic transmission in South Korea from February 2020 to April 2021, a period during which cases caused by COVID-19 variants were rarely reported. These estimates were used to obtain more accurate estimates of the time-dependent basic reproduction number $R_{t}$. This information is crucial for understanding the effectiveness of various interventions implemented during the COVID-19 pandemic and for planning future responses to infectious diseases. Our results found that the reproduction number based on the GI may result in the larger value of $R_{t}$, which is an indicator of the COVID-19 transmission potential during the rapid increase of cases. Because presymptomatic transmission can occur without any noticeable symptoms, the value of $R_{t}$ obtained through the generation interval is higher than that obtained using the serial interval. Consequently, we can infer that the reproduction number obtained through the generation interval is more precise. These findings provide crucial insights that can aid in our understanding of COVID-19 transmission and facilitate future research.

The serial interval is the time between onset of symptoms in a primary case and onset of symptoms in a secondary case that they infected. Because SI is easily observable, it is common to use SI as a proxy of the GI. SI is calculated using data from epidemiological investigations and information on symptomatic infected cases. This information is used to establish the time of infection for each case and the time interval between the infections of the primary and secondary cases. The serial interval is important in understanding the transmission dynamics of an outbreak and can be used to estimate the basic reproduction number $R_{t}$ and to predict the spread of the disease. On the other hand, the value of $R_{t}$ based on the generation interval may be larger than that obtained from the serial interval because it takes into account both confirmed symptomatic and presymptomatic infections as well as unconfirmed presymptomatic or asymptomatic infections. This makes the generation interval a more suitable parameter for predicting the number of infected people required for estimating immunity.

Moreover, this approach can be improved by considering the heterogeneity of sociodemographic variables and the generation interval of the presymptomatic, asymptomatic and symptomatic infectors. Since key parameters can vary depending on factors such as region, age, and gender, it may be possible to estimate parameters by considering these characteristics. For our future research, we can divide time periods for several outbreaks and apply the methods proposed in this study to compare the generation intervals, incubation periods, and the proportion of presymptomatic transmission including age, gender and regional characteristics. Additionally, it can be useful to incorporate the effects of asymptomatic infection and vaccination rate as well.

Our study has several limitations, one of which is that the estimates for the incubation period, generation time and serial intervals were not incorporated with the heterogeneity of age groups. Studies such as Kwok et al. (27) have shown that intra-age group transmission is more likely to occur than inter-age group transmission. Alene et al. (28) reviewed a total of 23 previous studies about the estimation of SI, which was between 4.2 and 7.5 days. Those studies described the estimated SI only but did not consider other key parameters. Another limitation is that the data was collected from February 2020 to April 2021 in Busan, Korea and thus the estimates for key parameters are specific to that region and time period. Jeon et al. (29) have estimated serial intervals according to different time periods related to control interventions in South Korea, but the present study focused on estimating key parameters during on-going outbreak before the emergence of COVID-19 variants in Korea. It is important to note that these estimates are based on data collected in one specific location and may not necessarily apply to other regions or at different stages of the pandemic. In conclusion, this work provides valuable information on the key epidemiological parameters of COVID-19 in South Korea. The estimates of incubation period, serial interval, generation interval and proportion of presymptomatic transmission are important to quantify the transmissibility and effects of various interventions. The results show that the reproduction number based on generation interval may result in the larger value of $R_{t}$, highlighting the importance of considering presymptomatic transmission and generation intervals when estimating the time-varying reproduction number. Our results provide important insights that can be used to improve our understanding of the spread of COVID-19 and inform future research.

## Data availability statement

The original contributions presented in the study are included in the article material, further inquiries can be directed to the corresponding author.

## Author contributions

TK and SK: analyzed the data. TK, HL, and SL: drafted and revised the manuscript. TK, HL, SL, CK, and HS: interpreted the results. All authors contributed to the article and approved the submitted version.
